# Profile measurement of concave spherical mirror and a flat mirror using a high-speed nanoprofiler

**DOI:** 10.1186/1556-276X-8-231

**Published:** 2013-05-16

**Authors:** Koji Usuki, Takao Kitayama, Hiroki Matsumura, Takuya Kojima, Junichi Uchikoshi, Yasuo Higashi, Katsuyoshi Endo

**Affiliations:** 1 Department of Precision Science and Technology, Graduate School of Engineering, Osaka University, 2-1 Yamadaoka, Suita, Osaka 565-0871, Japan; 2 Applied Research Laboratory, High Energy Accelerator Research Organization, Oho 1-1, Tsukuba, Ibaraki 305-0801, Japan; 3 Research Center for Ultra-Precision Science and Technology, Osaka University, 2-1 Yamadaoka, Suita, Osaka 565-0871, Japan

**Keywords:** Profiler, Direct measurement, Normal vector, Ultraprecision mirror, Aspheric mirror, Slope error, Five-axis simultaneous control, Ultraprecision machining

## Abstract

Ultraprecise aspheric mirrors that offer nanofocusing and high coherence are indispensable for developing third-generation synchrotron radiation and X-ray free-electron laser sources. In industry, the extreme ultraviolet (wavelength: 13.5 nm) lithography used for high-accuracy aspheric mirrors is a promising technology for fabricating semiconductor devices. In addition, ultraprecise mirrors with a radius of curvature of less than 10 mm are needed in many digital video instruments. We developed a new type of nanoprofiler that traces the normal vector of a mirror's surface. The principle of our measuring method is that the normal vector at each point on the surface is determined by making the incident light beam on the mirror surface and the reflected beam at that point coincide, using two sets of two pairs of goniometers and one linear stage. From the acquired normal vectors and their coordinates, the three-dimensional shape is calculated by a reconstruction algorithm. The characteristics of the measuring method are as follows: the profiler uses the straightness of laser light without using a reference surface. Surfaces of any shape can be measured, and there is no limit on the aperture size. We calibrated this nanoprofiler by considering the system error resulting from the assembly error and encoder scale error, and evaluated the performance at the nanometer scale. We suppressed the effect of random errors by maintaining the temperature in a constant-temperature room within ±0.01°C. We measured a concave spherical mirror with a radius of curvature of 400 mm and a flat mirror and compared the results with those obtained using a Fizeau interferometer. The profiles of the mirrors were consistent within the range of system errors.

## Review

Ultraprecise aspheric mirrors that offer nanofocusing and high coherence are indispensable for developing third-generation synchrotron radiation sources such as Super Photon ring-8, the European Synchrotron Radiation Facility, and the Advanced Photon Source. Toward the practical realization of these light sources, much scientific equipment and many analytical instruments that outperform conventional instrumentation are being designed. Hard X-rays at nanoscale spatial resolution are expected to find wide applications in areas such as nanotechnology, materials, biotechnology, medical treatment, and medical manufacture.

In industry, the extreme ultraviolet (wavelength: 13.5 nm) lithography used for high-accuracy aspheric mirrors is a promising technology for fabricating semiconductor devices. In addition, many digital video instruments require ultraprecise mirrors with a radius of curvature of less than 10 mm [1,2]. A light condensing or image optical system mirror in the hard X-ray and EUV regions must perform near the diffraction limit in order to apply these light sources, which have spatial resolutions on the order of nanometers. That is, a next-generation ultraprecision mirror must meet the following requirements: a surface roughness of 0.1 nm peak to valley (PV) and an accuracy of form of 0.2 nm RMS. It is essential that ultraprecision machining and measurement technology progress considerably to produce such a next-generation ultraprecision mirror. Moreover, the measurement techniques require higher precision than the machining methods.

Currently, these optical components are measured by interferometers and coordinate measuring machines (CMMs) [3,4]. A CMM can measure an aspheric surface. Their reported accuracy is extremely precise, which is 10 to 100 nm. CMMs perform contact-type measurement, although they rarely damage samples because of the low measurement pressure of 15 mgf. They can measure only up to an inclination angle of 60° because the probe approaches from the upper *Z*-direction and scans the surface shape. Therefore, they are unsuitable for the measurement of machine elements with a high aspect ratio. The phase shift Fizeau interferometer can measure an aspheric surface with a high accuracy of 30 nm. However, it has limitations; it requires an external optical reference and depends on its precision, and it cannot measure a mirror with a large radius of curvature. In addition, the measured object must be approximately at least 100 mm in size. Other profilers include the Nanometer Optical Component Measuring Machine at Helmholtz Zentrum Berlin/BESSY-II [5] and the Extended Shear Angle Difference instrument at the Physikalisch-Technische Bundesanstalt [6]. These profilers are currently the most widely used, and their measurement accuracy is equal to 0.5 μrad RMS (3 nm RMS). However, the measurement range is limited to ±5 mrad (the radius of curvature is ±500 m for a length of 100 mm), and they can measure only sectional two-dimensional shapes in a straight line. There is no way to measure an aspheric surface with an accuracy within the order of a nanometer.

The purpose of this study is to develop a direct, non-contact profiler to measure aspheric surfaces with a radius of curvature from flat to 10 mm, with a figure error of less than 1 nm PV, a slope error of less than 0.1 μrad, and a measurement time of less than 5 min/sample.

### Principle of measurement

**Figure 1:**
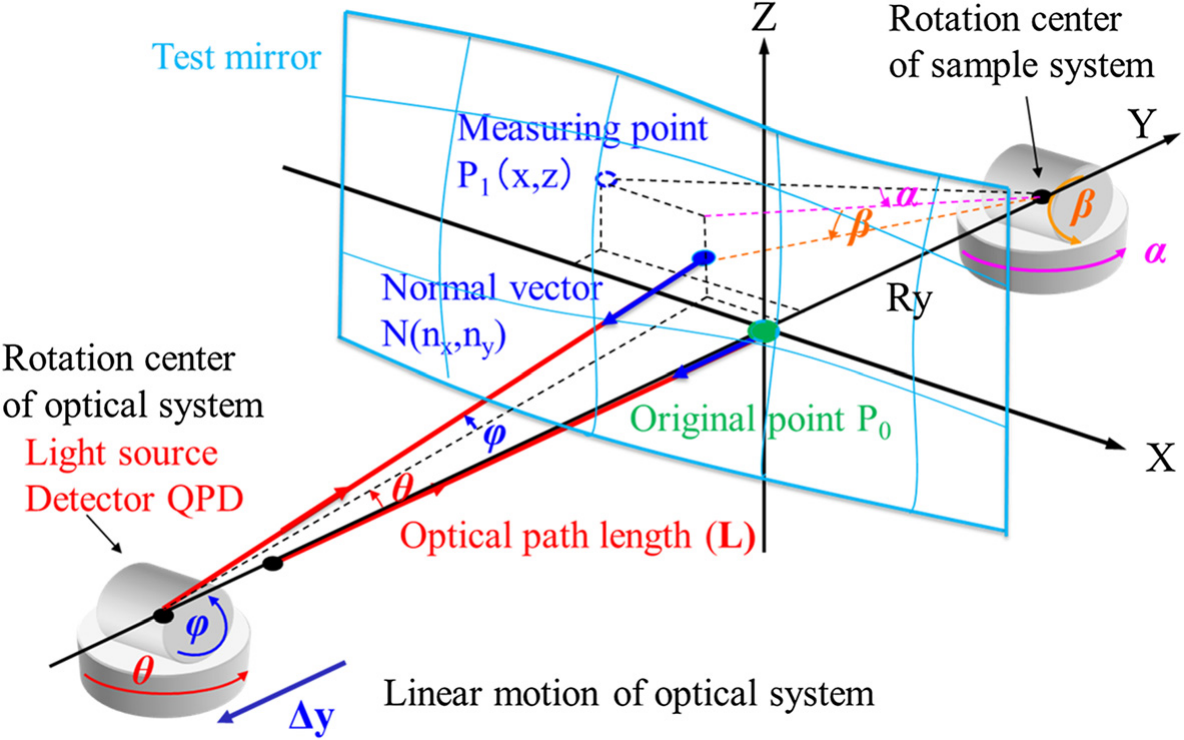
Principle of profile measurement by normal vector tracing.

Figure 1 illustrates the measurement principle of the profiler. This measuring method is based on the straightness of laser light and the accuracy of a rotational goniometer [7,8]. Detector quadrant photodiode (QPD) is established at the rotation center of two sets of goniometers at the optical system side; moreover, a light source is set at the position where it is equal to a rotation center optically, and a measured surface is assembled so that the distance becomes *R*_*y*_ from the original point of the measured surface to the rotation center of two sets of goniometers at the sample system side. The normal vectors of each point on the mirror surface are determined by making the incident light beam on the surface and the reflected beam at that point coincide, through the use of a straight stage (*Δy*) and two sets of goniometers (*θ, φ, α, β*), each consisting of a pair of goniometers. This method measures the normal vectors (*n*_*x*_*, n*_*z*_) and their coordinates (*x, z*) on the specimen surface using the straightness of a laser beam. The surface shape is obtained from the normal vectors and their coordinates using a reconstruction algorithm. The machine consists of an optical system with two goniometers and one linear motion stage and a specimen system with two goniometers [9,10].

Each normal vector on the specimen surface is equivalent to the light vector when the incident and reflected light paths coincide. To achieve this, the reflected beam is controlled to return to the center of the QPD using the motion of each stage. Then, each normal vector is determined from the angle of rotation of the goniometers. Moreover, during measurement, the optical path length (*L*) is kept constant by a *y*-stage (*Δy*), and the coordinates of each normal vector are determined.

**Figure 2:**
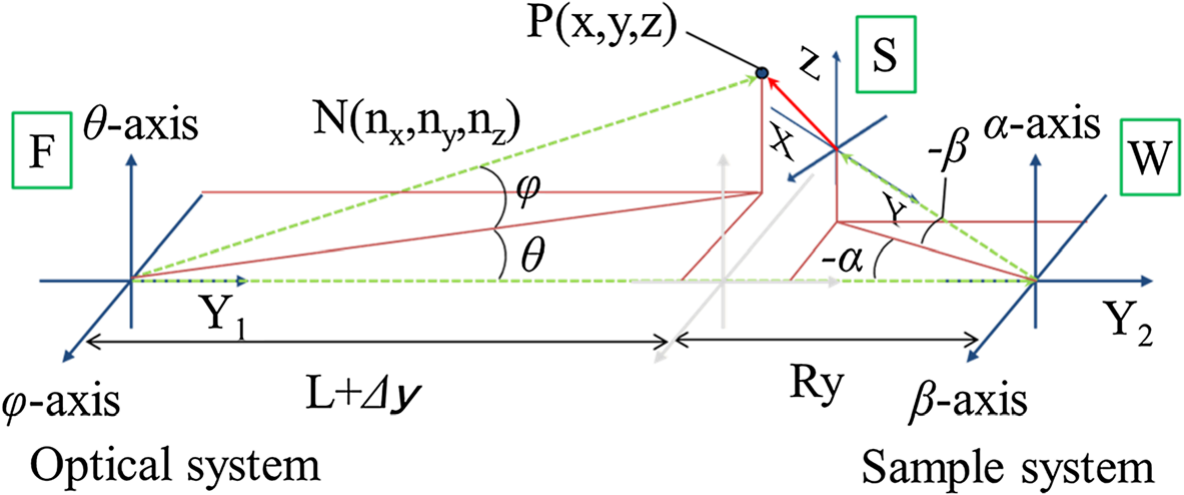
**Overall coordinate system in this measurement. ***F*, the coordinate system of the optical system. *W*, a coordinate system of the sample system. *S*, the coordinate system of the main body of sample.

Figure 2 shows the overall coordinate system in this measurement method. Measurement point coordinate *P* and normal vector *N* of the measured surface are values from coordinate system *S*. Therefore, firstly, measurement point coordinate *P* and the normal vector *N* are demanded in coordinate system *F*. It is made coordinate transformation in the order of *F* → *W* → *S*. In Figure 2, measurement point coordinate *P* and normal vector *N* are shown in Equations 1 and 2, in regard to coordinate system *F*.

(1) $$\begin{array}{l} P=\left(\begin{array}{c} x\\ y\\ z \end{array}\right)=\left(\begin{array}{ccc} \cos\theta & -\sin\theta & 0\\ \sin\theta & \cos\theta & 0\\ 0 & 0 & 1 \end{array}\right)\;\left(\begin{array}{ccc} 1 & 0 & 0\\ 0 & \cos\varphi & -\sin\varphi \\ 0 & \sin\varphi & \cos\varphi \end{array}\right)\;\left(\begin{array}{c} 0\\ L\\ 0 \end{array}\right)\\ \;=L\;\left(\begin{array}{c} -\cos\varphi \sin\theta \\ \cos\varphi \cos\theta \\ \sin\varphi \end{array}\right) \end{array}$$

(2) $$Ν=\left(\begin{array}{c} n_{x}\\ n_{y}\\ n_{z} \end{array}\right)=\left(\begin{array}{c} -\cos\varphi \sin\theta \\ \cos\varphi \cos\theta \\ \sin\varphi \end{array}\right)$$

Because there is the distance of coordinate system *F* and coordinate system *W* ‘*L*−Δ*y* + *R*_*y*_’ apart on *Y*_1_-axis, in regard to coordinate system *W,* measurement point coordinate *P* is expressed by the coordinate transformation that Equation 1 is translated. In regard to coordinate system *W*, normal vector *N* becomes the same as coordinate system *F*. Therefore, Equation 3 translated Equation 1, in regard to coordinate system *W*.

(3) $$P=\left(\begin{array}{c} x\\ y\\ z \end{array}\right)=L\;\left(\begin{array}{c} -\cos\;\varphi \sin\;\theta \\ \cos\;\varphi \cos\;\theta \\ \sin\;\varphi \; \end{array},\;\right)-\left(\begin{array}{c} 0\\ L-\mathrm{\Delta y}+\mathrm{Ry}\\ 0 \end{array}\right)$$

In regard to coordinate system *S*, when measurement point coordinate *P* and normal vector *N* are also translated, they become Equations 1 and 2, respectively.

(4) $$\begin{array}{l} P=\left(\begin{array}{c} x\\ y\\ z \end{array}\right)=\left(\begin{array}{ccc} 1 & 0 & 0\\ 0 & \cos\beta & \sin\beta \\ 0 & -\sin\beta & \cos\beta \end{array}\right)\;\left(\begin{array}{ccc} \cos\alpha & \sin\alpha & 0\\ -\sin\alpha & \cos\alpha & 0\\ 0 & 0 & 1 \end{array}\right)\\ \;\left[L\;\left(\begin{array}{c} -\cos\varphi \sin\theta \\ \cos\varphi \cos\theta \\ \sin\varphi \end{array}\right)-\left(\begin{array}{c} 0\\ L-\mathrm{\Delta y}+\mathrm{Ry}\\ 0 \end{array}\right)\right]+\left(\begin{array}{c} 0\\ \mathrm{Ry}\\ 0 \end{array}\right) \end{array}$$

(5) $$\begin{array}{l} N=\left(\begin{array}{c} n_{x}\\ n_{y}\\ n_{z} \end{array}\right)=\left(\begin{array}{ccc} 1 & 0 & 0\\ 0 & \cos\beta & \sin\beta \\ 0 & -\sin\beta & \cos\beta \end{array}\right)\;\left(\begin{array}{ccc} \cos\alpha & \sin\alpha & 0\\ -\sin\alpha & \cos\alpha & 0\\ 0 & 0 & 1 \end{array}\right)\\ \;\left(\begin{array}{c} -\cos\varphi \sin\theta \\ \cos\varphi \cos\theta \\ \sin\varphi \end{array}\right) \end{array}$$

Here, the shape derived by using *y* and *n*_*y*_ has low precision. Therefore, the shape is derived by assigning *P*(*x*, *z*) and ***N***(*n*_*x*_, *n*_*y*_) to derivation algorithm.

This profiler determines the surface shape from the normal vectors and their coordinates by rotational motion, which is more accurate than linear motion and requires no reference optics. Therefore, there are no limitations on the measured shape, and free-forms can be directly measured [11].

### Algorithm for obtaining the surface profile

We developed an algorithm for calculating the three-dimensional surface profile from the acquired normal vectors and their coordinates. A normal vector is equivalent to the surface slope or derivative of the surface profile. In this algorithm, to derive a figure from a normal vector and the coordinate, we express the figure by a model function and then fit the differential calculus function (slope function) to data on the normal vector by using the least-squares method. By calculating each coefficient of the series, the surface profile is determined. Equations 6 and 7 represent the surface shape and slope for the two-dimensional case, respectively; the same approach applies to the three-dimensional case.

(6) $$y\left(x\right)=\sum \left\{a_{n}•\cos\left(k_{0}\mathrm{nx}\right)+b_{n}•\sin\left(k_{0}\mathrm{nx}\right)\right\}$$

(7) $$\begin{array}{l} f\left(x\right)=\frac{\mathrm{dy}\left(x\right)}{\mathrm{dx}}\\ \;=\sum \left\{a_{n}•\left(-k_{0}n\right)•\sin\left(k_{0}\mathrm{nx}\right)+b_{n}•\left(k_{0}n\right)•\cos\left(k_{0}\mathrm{nx}\right)\right\} \end{array}$$

(8) $$\sum {∥f_{j}-f\left(x_{j}\right)∥}^{2}\to \min\mathrm{about}\left\{a_{n},b_{n}\right\}$$

(*f*_*j*_, normal vector or slope; *x*_*j*_, its coordinates).

### High-speed nanoprofiler

**Figure 3:**
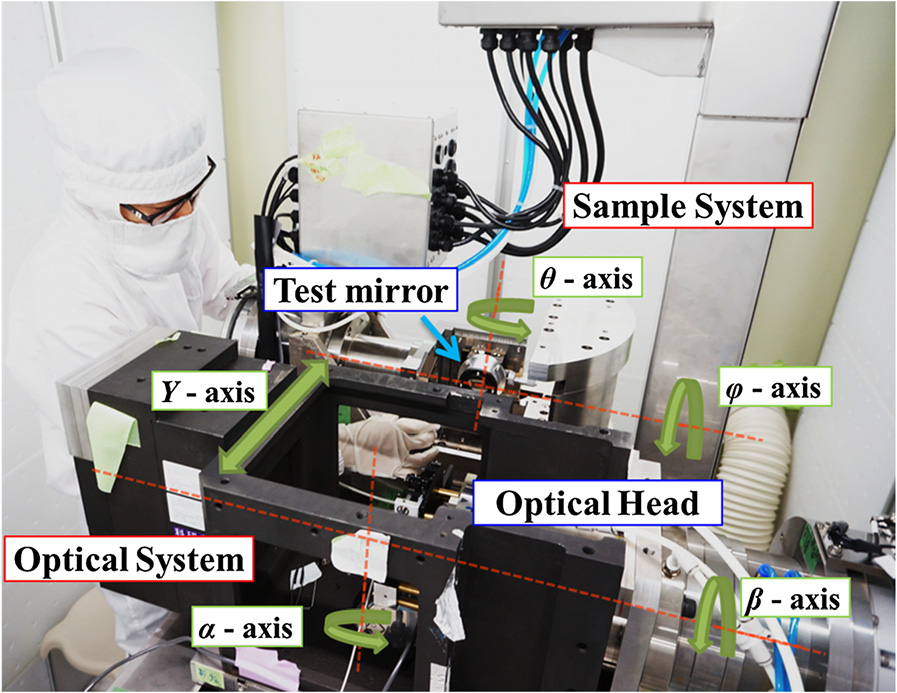
Photograph of newly developed nanoprofiler.

**Figure 4:**
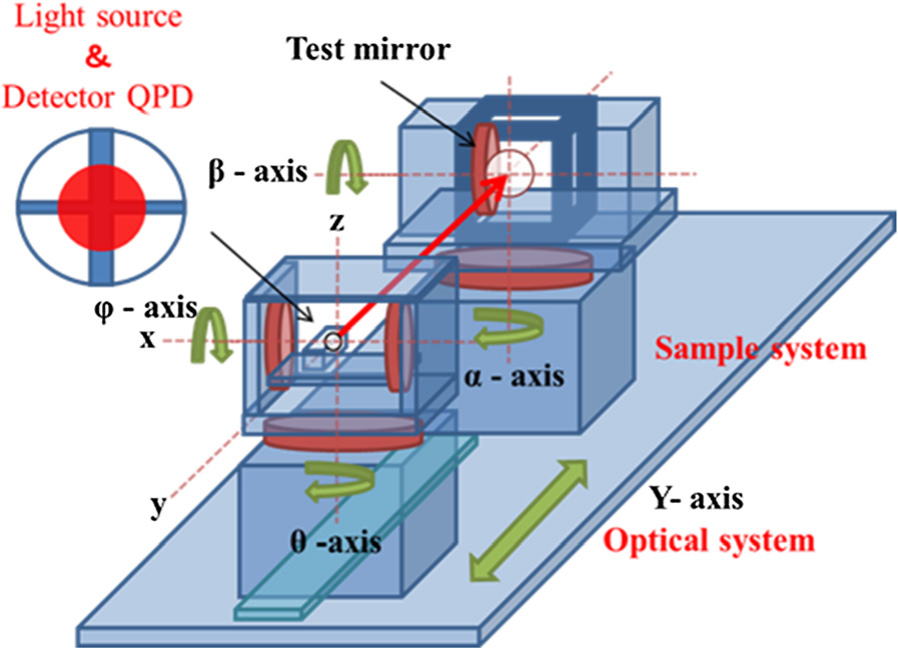
A schematic view of a nanoprofiler based on normal vector measurements.

Figures 3 and 4 show a photograph and a schematic view, respectively, of the newly developed nanoprofiler for normal vector tracing. The maximum mass of the main body of this machine is approximately 1,200 kg. The measurement sample can set up a greatest dimension to *Φ* = 50 mm × 40 mm, with a maximum mass of 1 kg and an optical pass length of 400 mm between the sample and the detector. Additionally, each optical element is set by the alignment that a laser beam changes 10 nm on QPD, when a normal vector changes 0.1 μrad. This machine has two pairs of two-axis rotational stages with resolutions of 0.17 μrad and one linear motion stage with a resolution of 1 nm. The laser beam from the light source scans the surface of the specimen. To return the reflected beam from the surface to the center of the QPD, each stage of the optical system is follow-up controlled. Ultimately, the normal vectors are acquired by each goniometer.

**Figure 5:**
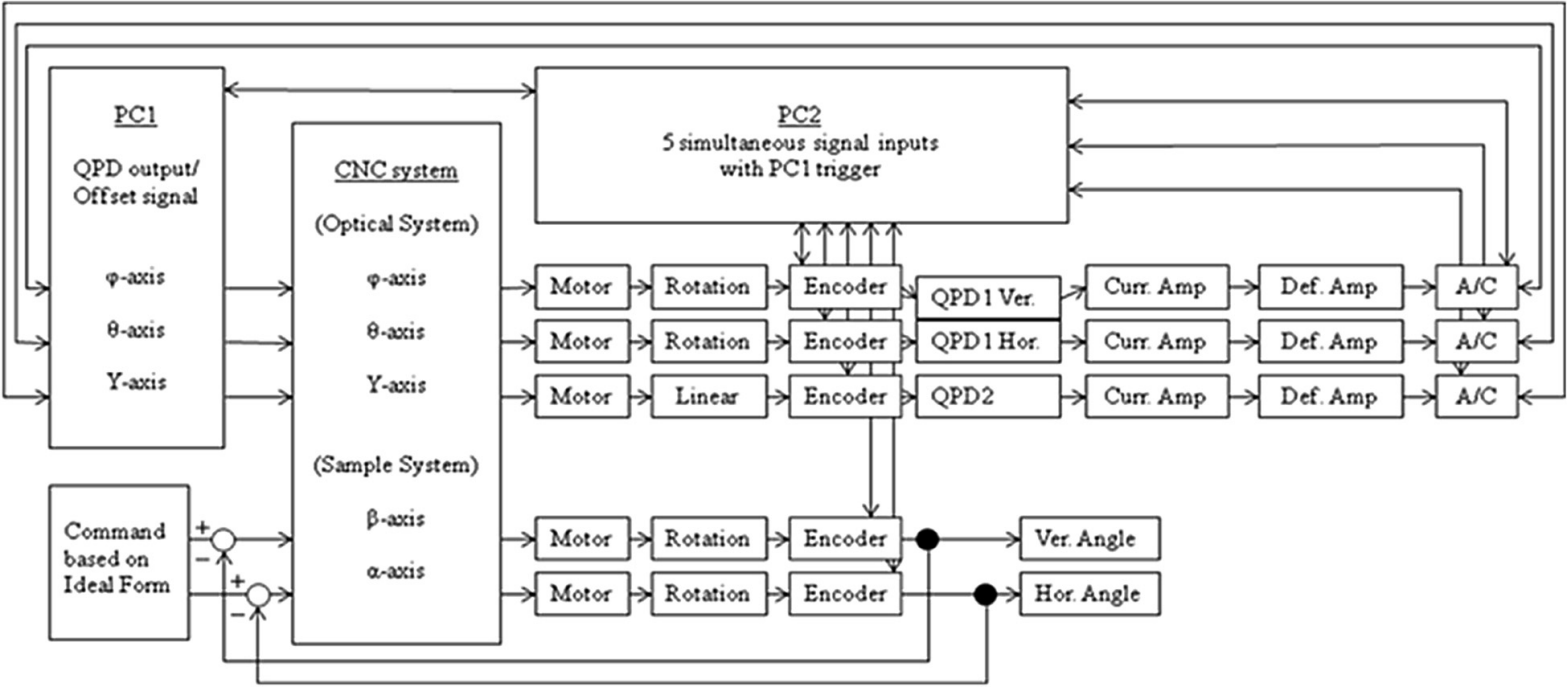
**Block-diagram of five-axis simultaneously controlling system.** The optical system with two rotational stages and one linear motion stage is follow-up controlled to trace normal vectors, while the sample system with two rotational stages is fixed-command controlled.

Figure 5 shows the five-axis simultaneous control system, which consists of an optical system and a sample system. The optical system has two rotational motions and one linear motion, which is follow-up controlled to trace the normal vectors. The sample system has two rotational motions, which are fixed-command controlled. This zero method in which the incident and reflected light paths are made to coincide avoids the effects of differences in QPD sensitivity and changes in the refractive index distribution. In fact, the stationary errors of normal vector tracing are larger than the target accuracy, so the QPD signal is read simultaneously with the output from the five-axis encoder. Consequently, the stationary errors can be ignored, and this process can be treated as the zero method.

### Measurement of a concave spherical mirror with 400 mm radius of curvature

We measured a concave spherical mirror with a 400 mm radius of curvature three times. The measurement time was 25 min. The optical system, i.e., the light source and QPD, was set at a point of 400 mm from the center of the mirror. When measuring a concave spherical mirror, if the optical system is set at the mirror's center of curvature, we do not need to move the sample system, and the reflected beam returns to the QPD within its dynamic range. Therefore, we can acquire normal vectors from the QPD output signal.

**Figure 6:**
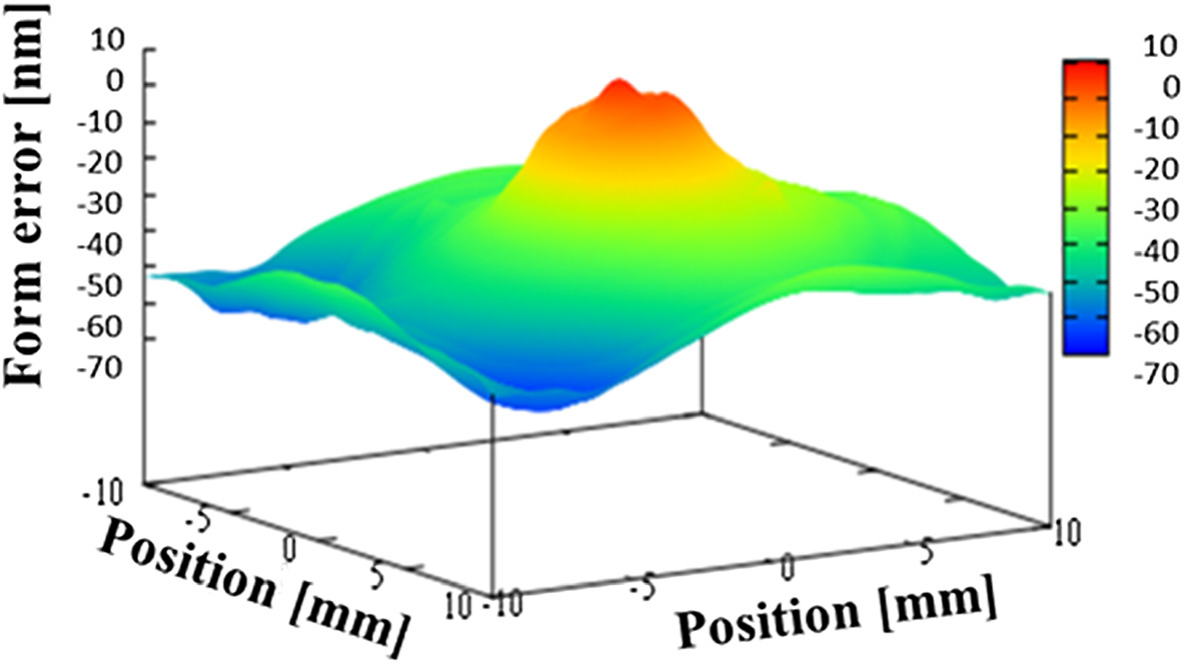
Figure error for concave spherical mirror (average of three measurements).

**Figure 7:**
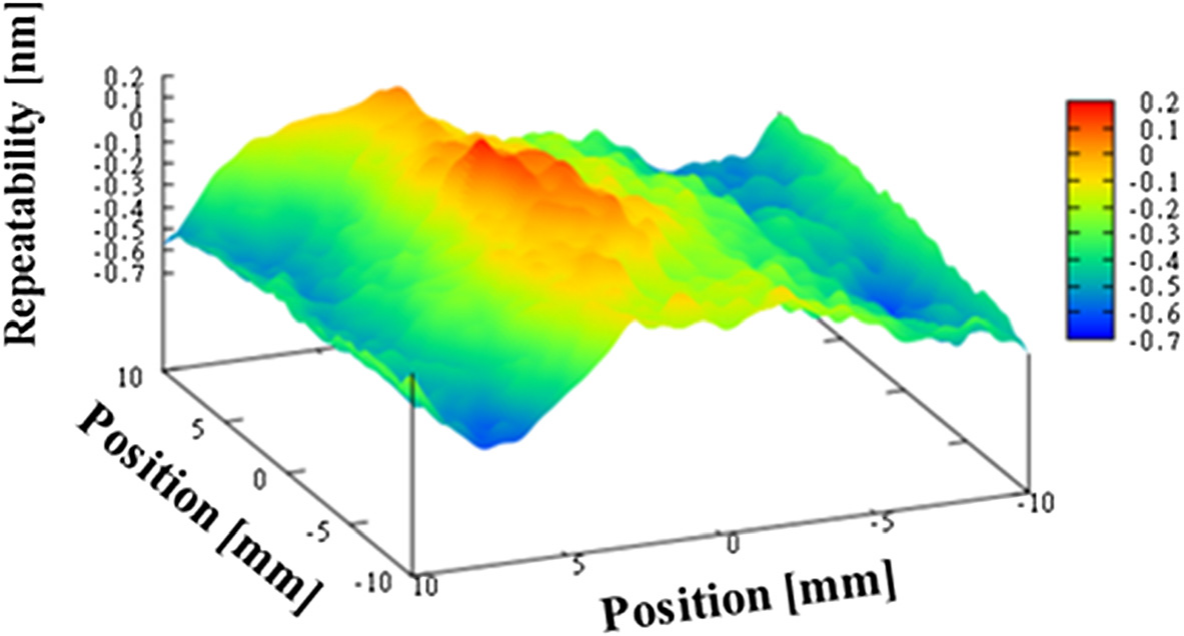
First-time repeatability for concave spherical mirror.

Figure 6 shows the average figure error for the three measurements, which is 70.5 nm PV. Next, we evaluated the repeatability. The repeatability is evaluated by taking the average of the shape error for three times, and finding a difference from the average. Figure 7 shows the first-time repeatability of our profiler. The repeatability was greater than 1 nm PV for all three measurements, as given in Table 1.

**Table 1:** Repeatability results for concave spherical mirror

	**First**	**Second**	**Third**
Repeatability	PV 0.81 nm	PV 0.74 nm	PV 0.85 nm

We can reduce random errors such as air flow and drift in temperature fluctuations by controlling the temperature, provided that we can further stabilize the constant-temperature room. The repeatability is expected to be improved by enhancing the environmental parameters to reduce these influences.

**Figure 8:**
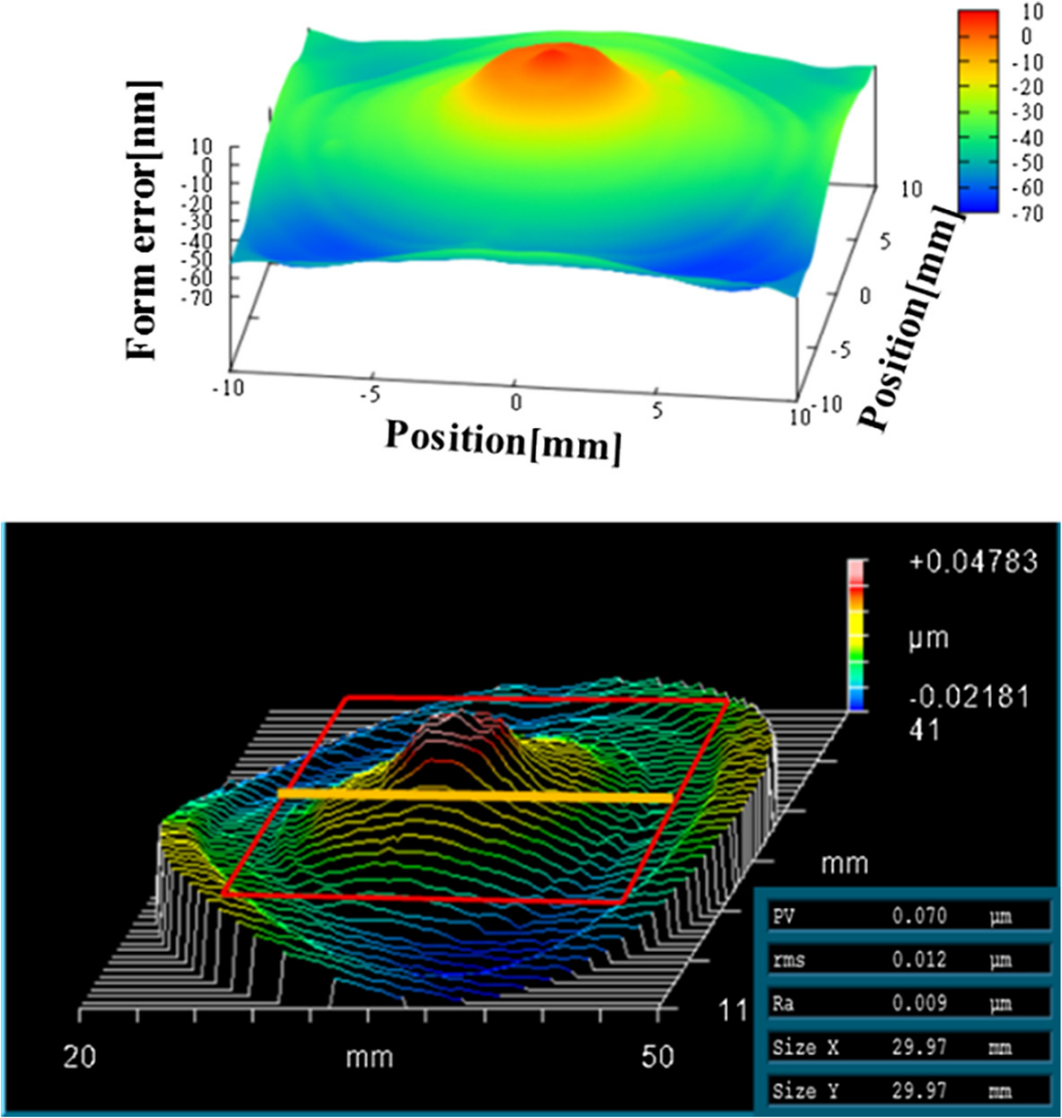
Fizeau interferometer results for concave spherical mirror in three dimensions.

**Figure 9:**
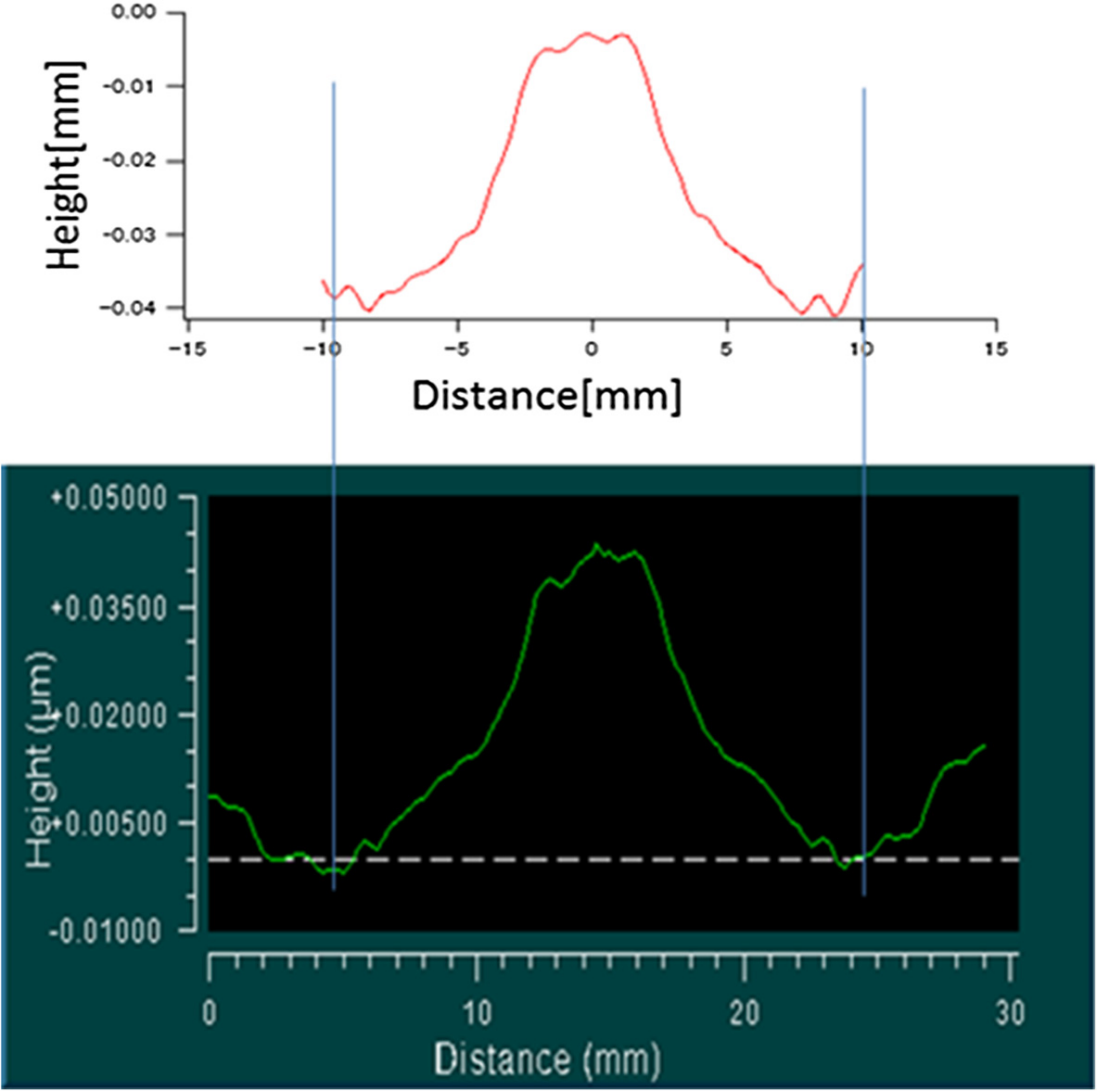
Fizeau interferometer results for concave spherical mirror in two dimensions.

In addition, we compared the results for the concave spherical mirror with those obtained using a Fizeau interferometer, as shown in Figures 8 and 9. The result for the Fizeau interferometer is 70.0 nm PV. Table 2 summarizes the results for both the profilers.

**Table 2:** Results of nanoprofiler and Fizeau interferometer for concave spherical mirror

	**Nanoprofiler**	**Fizeau interferometer**
In three dimensions	PV 70.5 nm	PV 70.0 nm
In two dimensions	PV 40.0 nm	PV 45.0 nm
Measurement range	20 × 20 mm	30 × 30 mm

The difference between the nanoprofiler and Fizeau interferometer results for the figure error may depend on each device's system error. On the other hand, the phase-shift Fizeau interferometer is affected by the precision of the reference mirror. We cannot conclude that the difference in these results is caused only by the greater precision of the nanoprofiler. Therefore, we conclude that the profiles of both the mirrors are consistent within the range of systematic error.

### Measurement of a flat mirror

We measured a flat mirror three times. The measurement time was 20 min. When measuring a flat mirror, we need to move the sample system which has two sets of two pairs of goniometers, optical system which has two sets of two pairs of goniometers and one straight stage, and the reflected beam returns to the QPD within its dynamic range. During the measurement, each axis is controlled numerically. The numerical control parameter is calculated in advance from the ideal shape of the sample. We detect the gap in the normal vector for the figure error using QPD because the sample has a figure error. Therefore, we can acquire the declination of the normal vectors from the QPD output signal.

**Figure 10:**
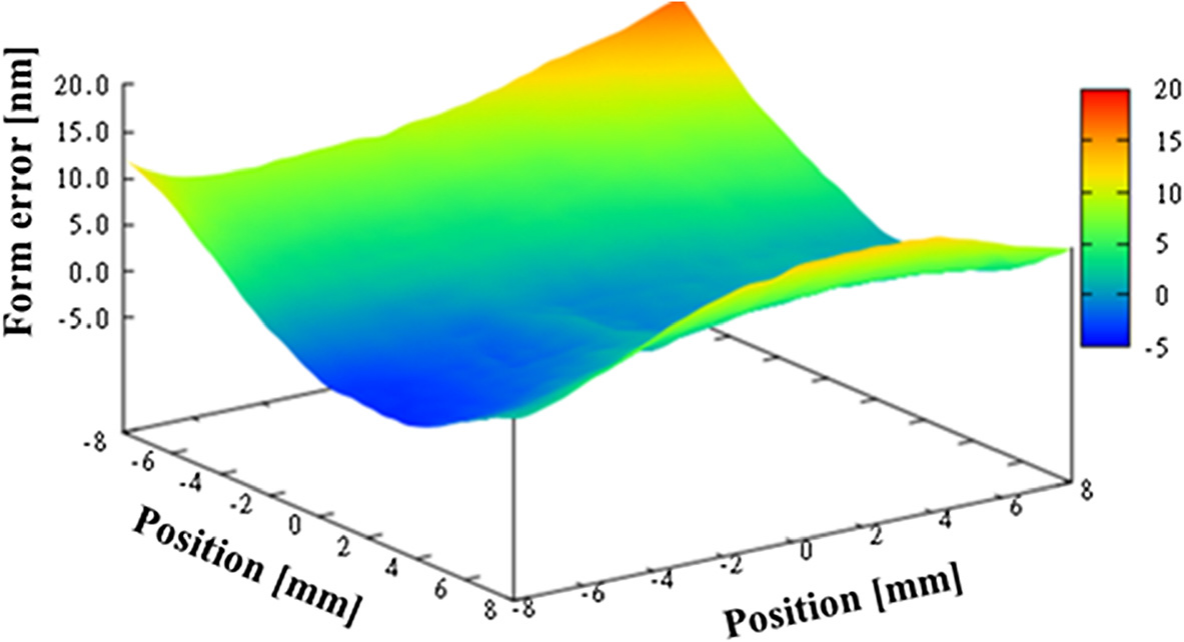
Figure error for flat mirror (average of three measurements).

**Figure 11:**
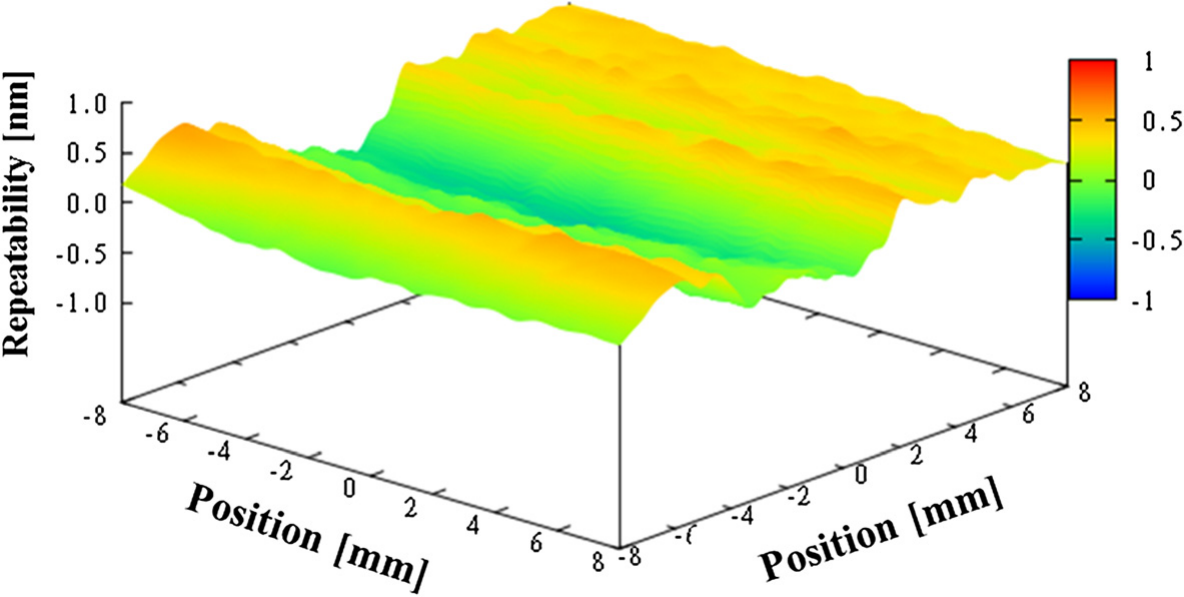
Repeatability for flat mirror (first time).

**Figure 12:**
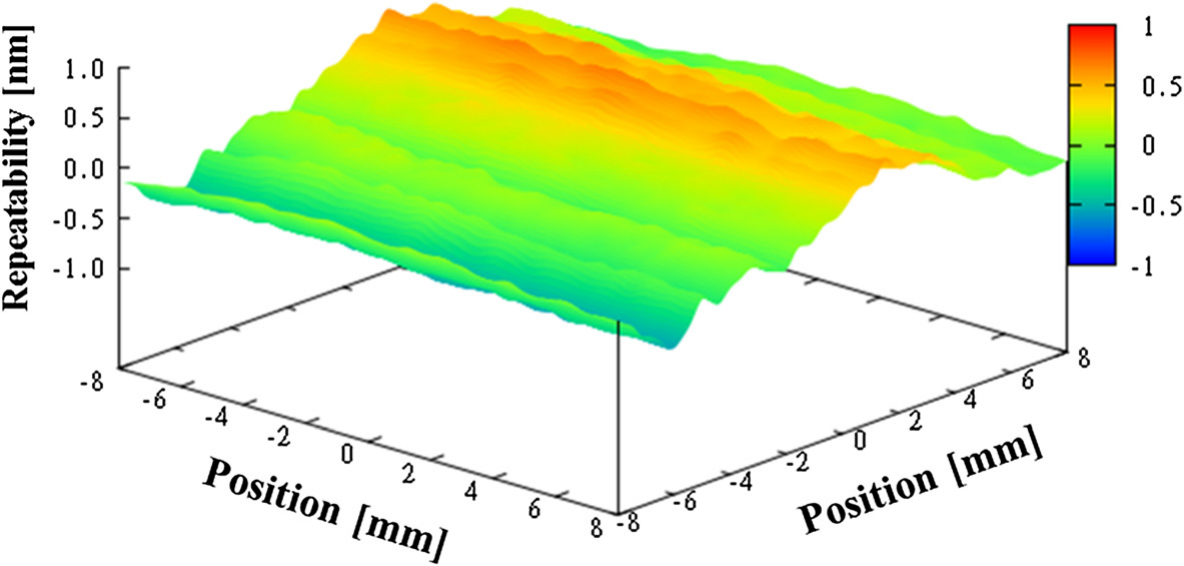
Repeatability for flat mirror (second time).

**Figure 13:**
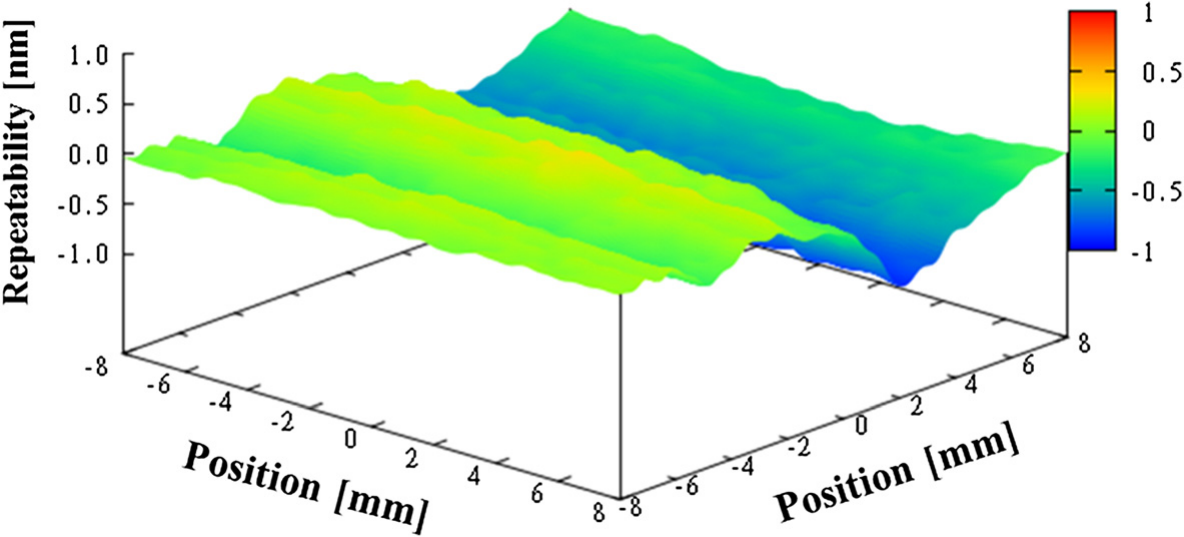
Repeatability for flat mirror (third time).

Figure 10 shows the average figure error for the three measurements, which is 21.0 nm. Next, we evaluated the repeatability of the measurements, as shown in Figures 11, 12, and 13. The repeatability of the first, second, and third measurements was 1.08 nm PV, 1.26 nm PV, and 1.25 nm PV, respectively.

When we compare the repeatability results, we see that the repeatability in each direction varies depending on the measurement. Because we used a raster scan method for these measurements, the acceleration and deceleration provided a rigorous method of measurement. Therefore, every measurement point is slightly different. The repeatability is expected to be enhanced by improving the dynamic stiffness of the optical head. In addition, when we measure a flat mirror, five axles are controlled and moved. Therefore, we cannot ignore the effect of the assembly error on the figure. If the assembly errors are evaluated, we expect to achieve measurements at an absolute shape precision of 1 nm PV by revising the systematic error in the future.

## Conclusions

In this study, we developed a high-speed nanoprofiler that uses normal vector tracing. This profiler uses the straightness of a laser beam and determines the normal vectors on a specimen's surface by acquiring the values of stages under five-axis simultaneous control. From each normal vector and its coordinates, the surface profile is obtained by a surface reconstruction algorithm.

To study the performance of the profiler, we measured a concave spherical mirror with a 400 mm radius of curvature and a flat mirror. For the concave spherical mirror, the repeatability was greater than 1 nm PV for all three measurements. In addition, we compared the results for the concave spherical mirror with those obtained using a Fizeau interferometer. The profile of the mirror was consistent within the range of the systematic error. For the flat mirror, the repeatability was almost 1.0 nm PV. To achieve our goal, the measurement method needs to be improved. If the assembly errors are evaluated, we expect to obtain measurements at an absolute shape precision of 1 nm PV by reducing the systematic error in the future.

## Competing interests

The authors declare that they have no competing interests.

## Authors’ contributions

KU carried out the measurements of the figure of the concave spherical mirror and the flat mirror, and drafted the manuscript. TK (Kitayama) developed an algorithm for reproduction of the figure from the normal vectors and the coordinates. HM designed the optical head. TK (Kojima) developed the data in the acquisition system. JU adjusted the system of the high-speed nanoprofiler. YH attached the concave spherical mirror and the flat mirror to the high-speed nanoprofiler and aligned them. KE conceived of the study and participated in its design and coordination. All authors read and approved the final manuscript.
